# *The Plant Cell* welcomes 2023 Assistant Features Editors

**DOI:** 10.1093/plcell/koac359

**Published:** 2022-12-15

**Authors:** Nancy A Eckardt, Blake C Meyers

**Affiliations:** Senior Features Editor, The Plant Cell, American Society of Plant Biologists, USA; Editor-in-Chief, The Plant Cell, American Society of Plant Biologists, USA; Donald Danforth Plant Science Center, St. Louis, Missouri 63132, USA; Division of Plant Sciences and Technology, University of Missouri-Columbia, Columbia, Missouri 65211, USA

**Figure 1:**
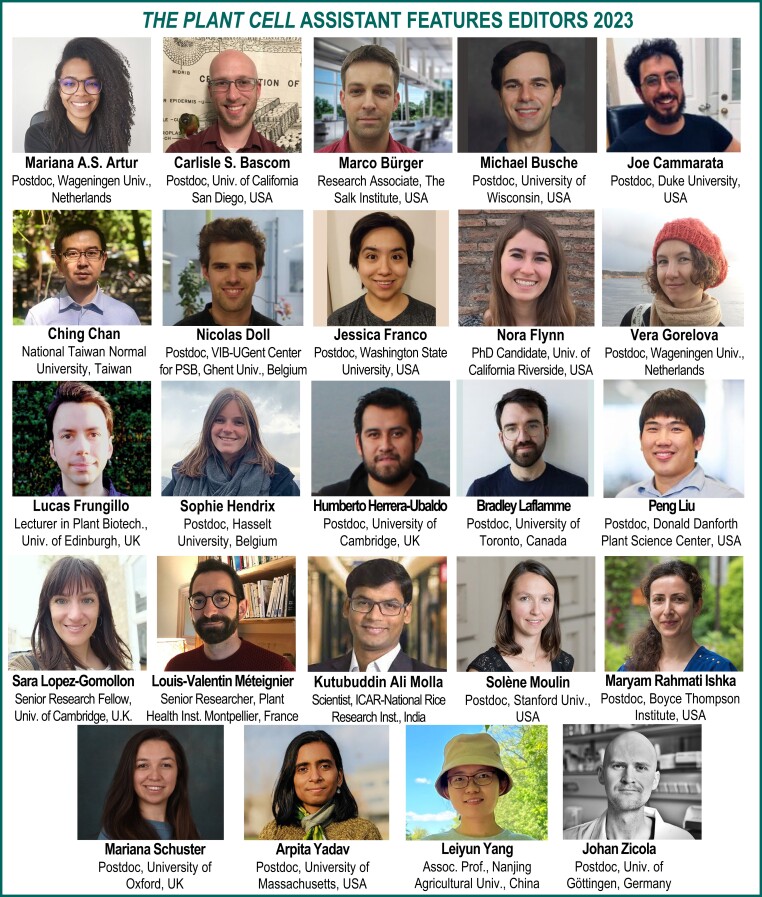
*The Plant Cell* AFEs for 2023.

*The Plant Cell* is pleased to announce our Assistant Features Editors (AFEs) for 2023 (Figure 1).

**Figure 2:**
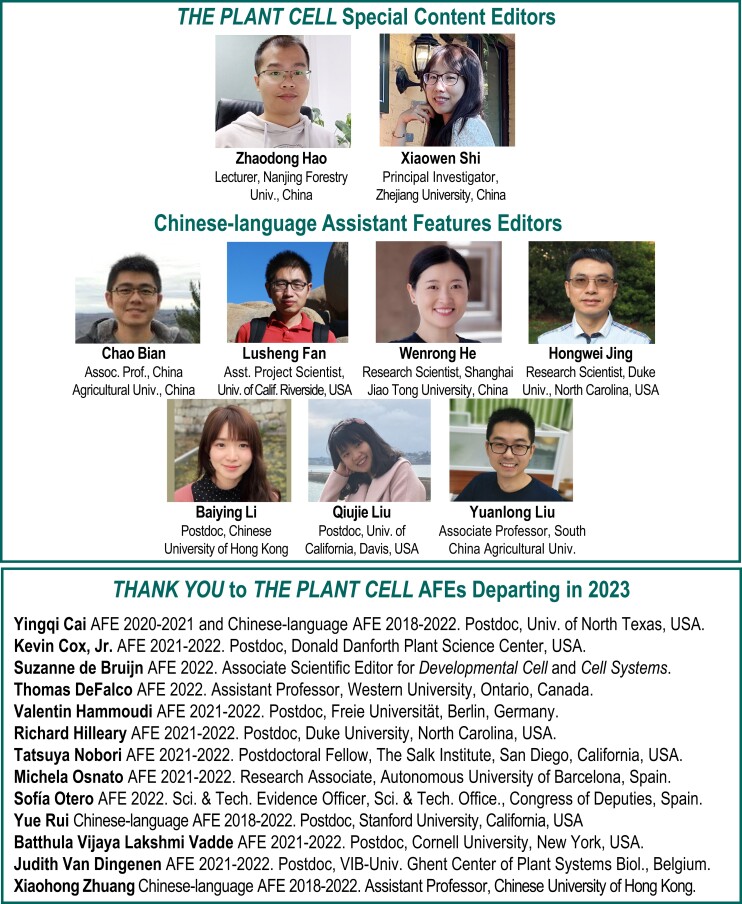
*The Plant Cell* Special Content Editors and Chinese-language AFEs for 2023 and AFEs departing in 2023.

The AFE program delivers a service to our authors by promoting their work published in *The Plant Cell*, serves our readers with these engaging short summaries of many of our research papers, and provides AFEs with valuable experience in writing for a broad audience, training in the peer review process, and networking opportunities. The AFEs contribute “In Brief” articles highlighting recent publications (in the front section of each monthly issue), gain experience and coaching to improve their writing, receive editor and peer-review training from Senior Editor mentors, participate in editorial board meetings, and engage in other journal-related activities. We look forward to working with another outstanding group of early career researchers in 2023. In addition, we thank our Special Content Editors, who manage *The Plant Cell* WeChat account, and our Chinese-language AFEs (Figure 2), who contribute translations of research article summaries in Chinese for WeChat.

We offer our sincere thanks to our departing AFEs for their service to the journal (Figure 2): Kevin Cox, Jr., Suzanne DeBruijn, Thomas DeFalco, Valentin Hammoudi, Richard Hilleary, Tatsuya Nobori, Michela Osnato, Sofía Otero, B. Vijaya Lakshmi Vadde, Judith Van Dingenen, and Chinese-language AFEs Yingqi Cai, Yue Rui, and Xiaohong Zhuang. We wish them all the best in their future endeavors.

*The Plant Cell* AFE program is highly competitive and a great addition to your C.V. Congratulations to all of our successful applicants! Our next round of applications will be announced in the summer of 2023 for AFEs to start in January 2024. Contact neckardt@aspb.org for more information.

